# Effect of repeated exposure to AQUI‐S^®^ on the viability and growth of *Neoparamoeba perurans*

**DOI:** 10.1111/jfd.12712

**Published:** 2017-09-25

**Authors:** R J Chance, Z Allcock, C J Secombes, B Collet, C Collins

**Affiliations:** ^1^ Scottish Fish Immunology Research Centre University of Aberdeen Aberdeen Scotland; ^2^ Marine Laboratory, Marine Scotland Aberdeen Scotland

**Keywords:** amoebic gill disease, anaesthesia, AQUI‐S^®^, isoeugenol, *Neoparamoeba perurans*

## Abstract

There have been recent efforts amongst immunologists to develop approaches for following individual fish during challenges with viral and bacterial pathogens. This study contributes to assessing the feasibility of using such approaches to study amoebic gill disease (AGD). *Neoparamoeba perurans*, agent of AGD, has been responsible for widespread economic and fish loss in salmonid aquaculture. With the emergence of AGD in Europe, research into infection dynamics and host response has increased. This study investigated the effect of repeat exposure to anaesthesia, a necessary requirement when following disease progression in individual fish, on *N. perurans*. In vitro cultures of *N. perurans* were exposed every 4 days over a 28‐day period to AQUI‐S^®^ (isoeugenol), a popular anaesthetic choice for AGD challenges, at a concentration and duration required to sedate post‐smolt salmonids. Population growth was measured by sequential counts of amoeba over the period, while viability of non‐attached amoeba in the culture was assessed with a vital stain. AQUI‐S^®^ was found to be a suitable choice for in vivo ectoparasitic challenges with *N. perurans* during which repetitive anaesthesia is required for analysis of disease progression.

## 1 INTRODUCTION

With the continued expansion of the global aquaculture industry (FAO, 2016), and related research on fish, there is a need for refinement in experimental approaches, including analyses of in vivo immune responses. A limited number of studies have been undertaken in developing methodologies such as individual monitoring (Collet et al., 2015; Monte, Urquhart, Secombes, & Collet, 2016; Urquhart et al., 2016). Benefits of such approaches include a reduction in the number of animals required for challenge experiments and higher quality data output, with reduced infection and response variability (Collet et al., 2015). While previous individual monitoring of fish following disease challenges has focused upon viral or bacterial pathogens, attention must also turn to parasite studies in view of serious parasite issues currently affecting aquaculture, for example sea lice and amoebic gill disease (AGD) affecting salmonid farming (Aaen, Helgesen, Bakke, Kaur, & Horsberg, 2015; Oldham, Rodger, & Nowak, 2016). To assess the suitability of this methodology for ectoparasites, *Neoparamoeba perurans,* the amoeboid aetiological agent of AGD, was selected as a model due to its recent emergence as a serious pathogenic threat to salmon aquaculture across northern Europe.

The first occasion of AGD as an epizootic was observed in an Atlantic salmon, *Salmo salar*, and rainbow trout, *Oncorhynchus mykiss*, sea farm located in east Tasmania, during the summer of 1984–85 (Munday, 1986). It was suggested that the aetiological agent of AGD could be classified as the normally free‐living *Neoparamoeba* sp. amoebae (Roubal, Lester, & Foster, 1989).

However, failed attempts to induce AGD in laboratory exposures with cultured *Neoparamoeba pemaquidensis* brought the validity of the causative agent under question (Kent, Sawyer, & Hedrick, 1988; Morrison, Crosbie, Cook, Adams, & Nowak, 2005). Young, Crosbie, Adams, Nowak, and Morrison (2007) were able to determine the true aetiological agent of AGD, as a newly described species *Neoparamoeba perurans*, which has since been cultured in vitro and used to fulfil Koch's postulates (Crosbie, Bridle, Cadoret, & Nowak, 2012).

To date, AGD is the most important disease associated with salmonid aquaculture in Australia, with reported losses of 10%–20% annually in addition to (freshwater bathing) treatment costs (Munday, Zilberg, & Findlay, 2001). Further cases of AGD in marine farmed Atlantic salmon, the most susceptible species to the disease, have been reported in Chile, with a 55.7% disease prevalence in the summer 2007–2008 (Bustos et al., 2011; Rozas, Bohle, Grothusen, & Bustos, 2012); Canada (ICES, 2015); France; and Spain (Munday et al., 2001; Rodger & McArdle, 1996) and a land‐based partial recirculation system in South Africa during 2009–10 (Mouton, Crosbie, Cadoret, & Nowak, 2014). In recent times, Northern Europe has suffered increasing AGD prevalence with substantial economic and fish stock losses. It was first described in eight farms in Ireland, in 1995 (Palmer, Carson, Ruttledge, Drinan, & Wagner, 1997; Rodger & McArdle, 1996), then in Scotland, United Kingdom in 2006 (Young, Dyková, Snekvik, Nowak, & Morrison, 2008), with typical losses ranging from 10% to 20% but occasionally reaching 70% (Marine Scotland 2012). In 2011, >25% of salmon aquaculture sites in Ireland and Scotland reported AGD, with economic losses estimates at USD$81M (Rodger, 2014; Shinn et al., 2015). Norwegian aquaculture has seen mortalities ranging between 12% and 82%, and outbreaks have increased from 5 in 2012, 56 in 2013 to 70 in 2014 (Powell, Reynolds, & Kristensen, 2015; Steinum et al., 2008).

The recognized method of obtaining pathogenic samples of *N. perurans* is to collect specimens from the gills of infected fish at the point of lethal sampling (Morrison, Crosbie, & Nowak, 2004), which involves at least one exposure to fish anaesthetic. Recent work from Shijie, Adams, Nowak, and Crosbie (2016) has demonstrated that a single exposure to anaesthetics containing eugenol did not inhibit population growth or attachment abilities of cultured *N. perurans*.

To develop a non‐lethal sampling approach requires repeated anaesthesia of fish, which in turn, for an ectoparasitic disease model such as AGD, also results in repeated anaesthesia of the pathogen. Therefore, the first step in developing a non‐lethal challenge model for AGD is to examine the effect of repeat exposure of *N. perurans* to anaesthesia.

AQUI‐S^®^ is a gel‐like anaesthetic that was first developed in New Zealand in 1996. Inspired by the anaesthetic capabilities of clove oil (eugenol), AQUI‐S^®^ contains as active ingredient 50% isoeugenol (not present in natural clove oil) and 50% emulsifier polysorbate 80 (Javahery & Moradlu, 2012). It is the only registered food‐grade anaesthetic with zero withdrawal time in Australia, Chile, Costa Rica, Honduras, Korea, and New Zealand; (AQUI‐S^®^, 2015). As of 2014, AQUI‐S^®^ has also been approved in Norway for sedation and anaesthesia of Atlantic salmon and rainbow trout prior to and during handling events, and in live fish transport (Kolarevic & Terjesen, 2014). AQUI‐S^®^ was therefore selected due to the popularity of use in countries most severely affected with AGD, alongside recent findings of no short‐term impacts upon attachment or viability of *N. perurans* after single exposure (Shijie et al., 2016). This is the first paper to report upon the repeated exposure of *N. perurans* to fish anaesthetics and to describe any adverse effects found on this aquaculture ectoparasite.

## 2 MATERIALS AND METHODS

### 2.1 Preparation of flasks

A polyclonal culture of *N. perurans*, isolated and maintained at Marine Laboratory, Marine Scotland Science as described in Collins et al. (2016), was used in experiments. The concentration of amoeba present in a pooled seawater overlay of *N. perurans* in vitro cultures was estimated as follows. Four 100 μl aliquots of amoeba culture from the pooled culture overlay were added to a 96‐well plate (Greiner GMH) and three technical replicate 10‐fold dilutions made from each initial aliquot. The amoebae were allowed to settle in wells for 20 min and then counted with an inverted microscope. Means of the counts, adjusted for dilution factor, were calculated and used to estimate the number of amoebae in the pooled overlay.

Twelve 25‐cm² tissue culture flasks (Greiner) with a malt‐yeast agar [MYA; 0.01% (w/v) malt extract, 0.01% (w/v) yeast extract, 2% (w/v) bacteriological agar (Oxoid Ltd, UK)] under layer and a 7 ml 35ppt 0.22‐μm‐filtered (Steritop™ 0.22‐μm polyethersulfone (PES) membrane filters; Merck Millipore, Fisher Scientific) seawater overlay were inoculated with approximately 1,500 amoebae/ml. Cultures were stored in a 13°C incubator, and amoebae were left to adhere overnight.

### 2.2 Anaesthetic exposures

Six replicate flasks were used for each treatment: AQUI‐S^®^ (isoeugenol) (AQUI‐S New Zealand Ltd.) and control (35 ppt 0.22‐μm‐filtered sea water). Anaesthetic treatment flasks were exposed to the same concentrations and durations required to anaesthetize post‐smolt salmonids to Stage 4 anaesthesia, AQUI‐S^®^ at 17 mg/L for 20 min. Due to the small amounts of anaesthetic required, at each time point a stock solution was freshly prepared. An appropriate volume was dissolved in 35 ppt 0.22‐μm‐filtered sea water, pipetted into the 7 ml seawater overlay to obtain the required concentration, and the flasks agitated to ensure an even distribution of the anaesthetic across the culture.

After the predetermined exposure duration, the overlay containing the anaesthetic and the floating‐form amoeba was transferred to 15‐ml tubes, and the attached amoeba remaining in the flasks were rinsed once with filtered sea water and 6.9 ml of filtered sea water was provided to restore the overlay. Sea water used in the rinse was discarded. The original overlay containing anaesthetic and floating‐form amoeba was centrifuged at 1073×g for 10 min—it should be noted that this was additional contact time for the suspended amoeba population with AQUI‐S^®^—the supernatant removed and the amoebae present in the pellet transferred to a 1.5‐ml Eppendorf tube containing 1 ml filtered sea water. The amoeba suspensions were centrifuged at 11337×g for 1 min followed by the removal of the supernatant and resuspension of amoeba in 1 ml filtered sea water. Amoebae were washed a further time as above then returned to their respective flasks after being resuspended in 100 μl filtered sea water, returning the total overlay volume to 7 ml. Preliminary work utilizing the vital stain Neutral Red (Sigma‐Aldrich, N7005) ascertained that the speed of centrifugation and transfer had no negative effect upon the morphology or viability of the amoeba (albeit not amoebae exposed to anaesthetic) and that the speed and duration of centrifugation were sufficient to pellet the suspended amoeba from the suspension (data not shown). This process was also carried out for all control flasks at each time point. All flasks were returned to 13°C until the next scheduled exposure. Flasks were treated with anaesthetic every 4 days for a 28‐day period.

### 2.3 *Neoparamoeba perurans* population growth assessment

#### 2.3.1 Attached amoebae

Prior to seeding with amoebae, a transect was drawn diagonally across the bottom of each culture flask with five indents, spaced at 1‐cm intervals, extending from the top left corner to the middle of the flask to standardize the position in flasks where amoebae counts were obtained, and to help account for any potential differences between different flask areas in amoebae settlement and growth. Photographs of field of view were taken with an inverted microscope at ×10 magnification at each indent (*n* = 5) prior to each anaesthetic exposure time point and attached amoebae counted from photos. Attached amoebae counted were assumed alive due to their ability to attach and their morphology.

#### 2.3.2 Suspended amoebae

**Figure 1:**
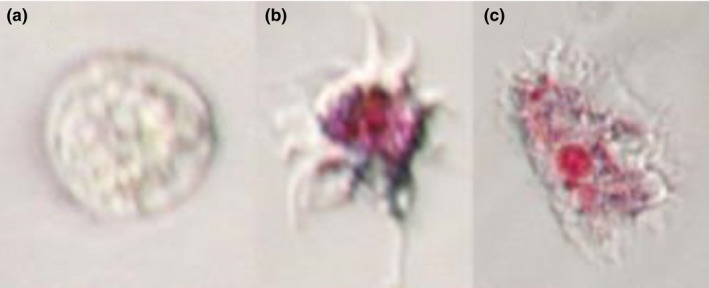
(a) Non‐viable *N. perurans* trophozoite after exposure to Neutral Red vital stain, showing characteristic spherical shape and absence of dye inclusions. (b) Viable floating trophozoite after Neutral Red exposure, with dye inclusions within lysosomes. (c) Viable attached amoeba after Neutral Red exposure

For the viability assessment of amoebae in suspension, a 200 μl aliquot of the seawater overlay was removed from each flask prior to each anaesthetic exposure time point; the seawater overlay of each flask was gently agitated for approximately 5 s and the flask rotated to an upright position so that the overlay pooled into the bottom left corner of the flask to ensure the aliquot obtained was representative of the total overlay. This aliquot was then transferred to a 1.5‐ml Eppendorf tube containing 4 μl of the vital stain Neutral Red. The tubes were kept at 13°C for 40 min to allow the amoebae to take up the stain. The cell suspensions were the centrifuged down for 1 min at 11337×g and the supernatant removed. The amoeba pellets were next resuspended in 100 μl filtered sea water and 10 μl of this suspension transferred to a well of a flat‐bottomed 96‐well plate containing 90 μl sea water. Amoebae were left to settle in the wells for 40 min and then were assessed for their viability with an inverted microscope at ×20 magnification. Viable amoebae had a diverse morphology as well as obvious dye inclusions, while non‐amoebae had no visible dye inclusions and a spherical morphology (Figure 1). Viable and non‐viable amoebae were counted.

#### 2.3.3 MS‐222 and metomidate flasks

The study also sought to assess the population growth and viability of *N. perurans* following repeated doses of powder‐based fish anaesthetics metomidate (12.5 mg/L) (AquaCalm™ Western Chemical Inc.) and MS‐222 (80 mg/L) (Sigma‐Aldrich). These treatments were carried out following the same methodology as detailed above for the AQUI‐S^®^ flasks. Due to the short exposure duration required for in vivo sedation, three and five minutes, respectively, the additional ten‐minute exposure of the suspended amoeba during the 1073×g centrifugation of the anaesthetic‐containing overlay and wash stage renders the total exposure time for these suspended populations ×4 and ×3 longer than required for a non‐lethal sampling procedure, and thus, these results should be interpreted with caution.

### 2.4 Statistical analysis

Population growth data were analysed with the statistical software package R (R Core Team, 2016). Total population count was constructed by combining mean count per field of view of attached amoeba and mean count of both viable and non‐viable amoeba in the 200 μl aliquot of seawater overlay. Data concerning attached population growth were subset into respective time points and analysed with a generalized linear mixed‐effects model (GLMM) with Poisson errors, with “Field Of View” as a fixed effect and “Flask” treated as a random effect to account for overdispersion, utilizing the statistical package “lme4” (Bates, Maechler, Bolker, & Walker, 2015). Data concerning suspended population counts were treated as above, minus the absent “Field of View” additions. Suspended amoeba percentage data were subset into respective time points and analysed with a linear model.

## 3 RESULTS

### 3.1 AQUI‐S^®^ population growth analysis

**Figure 2:**
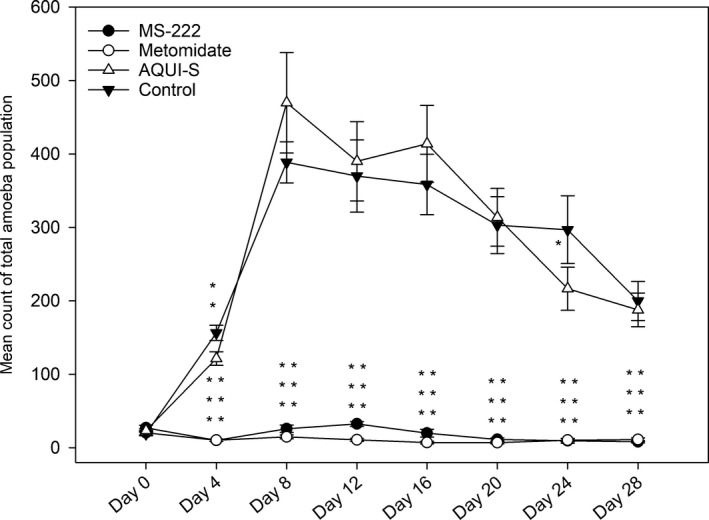
Mean counts of total amoeba populations (attached + suspended, viable + non‐viable). Totals were constructed from mean field of view of attached amoeba counts and numbers of suspended amoebae isolated in 200 μl aliquots taken from seawater overlay of the cultures. Where significance asterisks for both MS‐222 and metomidate are parallel, MS‐222 asterisks are represented on the left and metomidate on the right. Data are means ± *SE*. * *p* < .05, ** *p* < .01, *** *p* < .001

**Figure 3:**
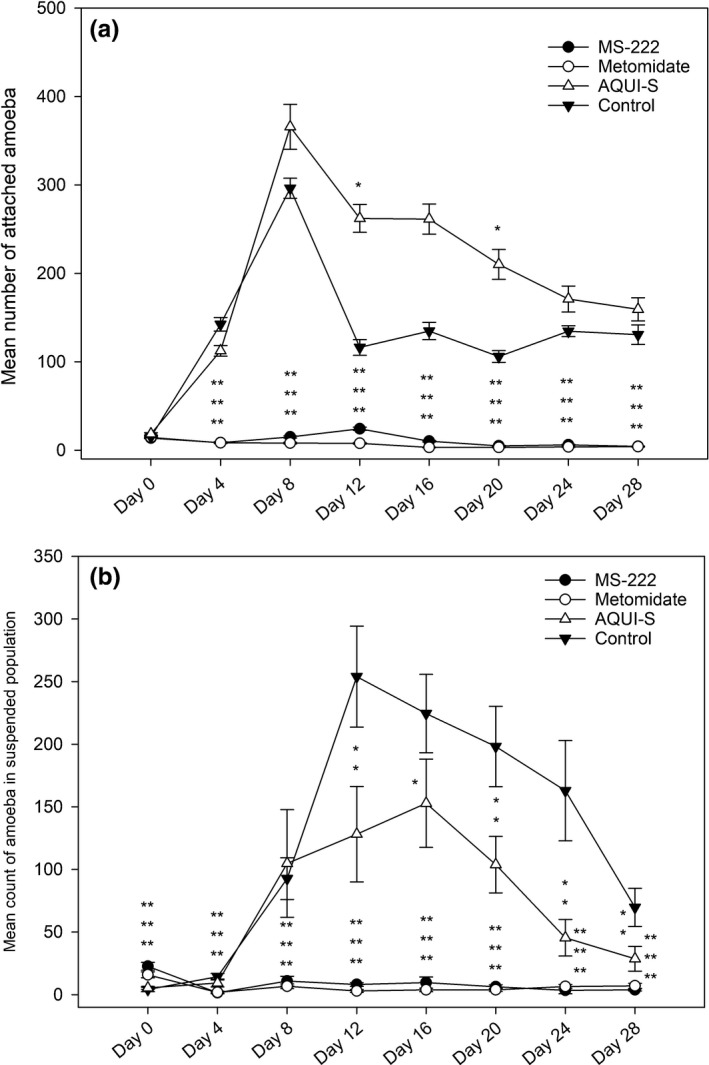
(a) Mean counts of attached amoeba constructed from mean field of view. (b) Mean count of total amoeba present in the suspended amoeba population isolated in 200 μl aliquots taken from seawater overlay of the cultures. Data are means ± *SE*. *p < .05, **p < .01, ***p < .001

There were no statistically significant differences between the total (attached and suspended) population growth of the AQUI‐S^®^‐treated amoebae and the control amoebae for the duration of the experiment, aside from day 4 (*p* ≤ .01) and day 24 (*p* ≤ .05) when the AQUI‐S^®^‐treated flasks had significantly lower total amoebae populations when compared to control flasks (Figure 2). When assessing the total attached population growth, AQUI‐S^®^ treatment showed significantly higher (*p* ≤ .05) populations of attached amoeba on days 12 and 20 (Figure 3) compared to the control. In contrast to this, on days 12–28, AQUI‐S^®^‐treated flasks had significantly lower populations of amoeba in suspension when compared to the controls (Figure 3).

### 3.2 AQUI‐S^®^ non‐viable population analysis

**Figure 4:**
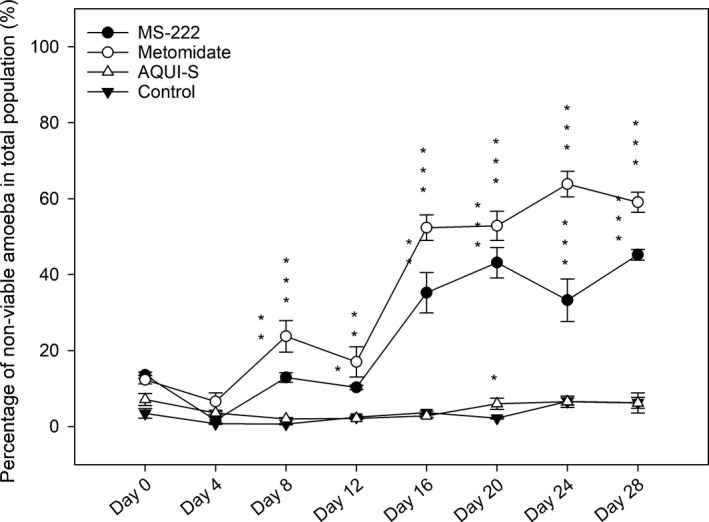
Mean percentage of non‐viable amoebae present in the total amoeba population calculated from mean field of view counts of attached amoeba and suspended amoebae isolated in 200 μl aliquots taken from seawater overlay of the cultures. Data are means ± *SE*. * *p* < .05, ** *p* < .01, *** *p* < .001

**Figure 5:**
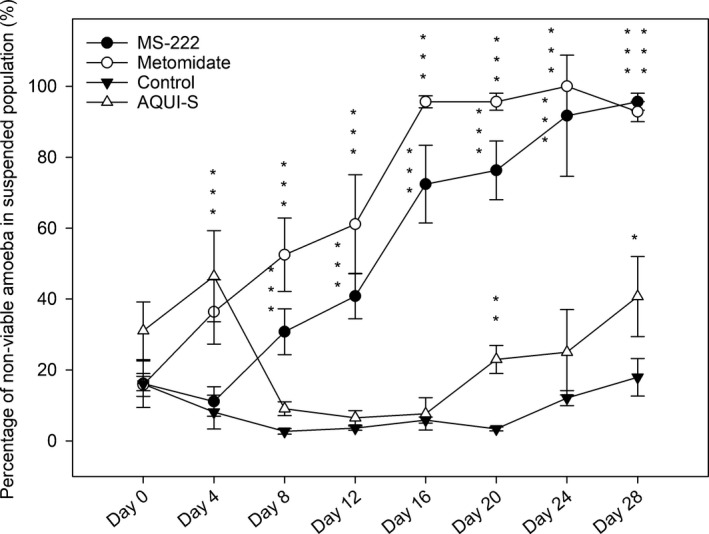
Mean percentage of non‐viable amoeba present in the suspended amoeba population isolated in 200 μl aliquots taken from seawater overlay of the cultures. Data are means ± *SE*. * *p* < .05, ** *p* < .01, *** *p* < .001

No statistical difference was seen in non‐viable amoebae percentage, in relation to the total amoebae population, in the AQUI‐S^®^ treatment when compared to the control, with the exception of day 20 (*p* < .05) where higher numbers of non‐viable amoebae were found in the AQUI‐S^®^ treatment (Figure 4). When considering percentage of non‐viable amoebae in only the suspended amoeba population, AQUI‐S^®^ flasks had significantly higher percentages of non‐viable amoebae on days 4, 20 and 28 when compared to control flasks (Figure 5).

### 3.3 MS‐222 and metomidate population growth analysis

From Day 4, total population counts for both MS‐222 and metomidate treatments were significantly lower (*p* ≤ .001) when compared to controls (Figure 2). Total amoebae population counts for both remained significantly lower than control flasks throughout the rest of the experiment. Attached amoebae populations in the MS‐222 and metomidate treatments mirror that of the total population counts; from day 4 onwards, with counts in both treatments remaining significantly lower (*p* ≤ .001) when compared to controls (Figure 3).

Following the first dose of anaesthetics (day 4), the populations of suspended amoebae mirror those of the attached populations, that is an initial reduction and statistically significant decrease in suspended population counts in comparison with the suspended amoebae in control and AQUI‐S^®^ flasks, with no recovery in numbers throughout the rest of the experiment. Prior to exposure to their respective anaesthetics at Day 0, both MS‐222 and metomidate flasks were found to have significantly higher (*p* ≤ .001) amoebae in suspension compared to the AQUI‐S^®^ and control (Figure 3). There were no significant differences found at Day 0, with respect to attached amoebae numbers, between any of the treatments and control (Figure 3). All amoebae were left to adhere overnight before treatment at Day 0, at the same temperature, in the same incubator in the agar base and seawater overlay derived from the same stocks. Therefore, it is not possible to suggest a lack of adherence by the amoeba due to culture differences in these treatments.

### 3.4 MS‐222 and metomidate non‐viable population analysis

After the second dose of anaesthetics (day 8), the percentage of non‐viable amoebae in both total and suspended populations in MS‐222 and metomidate flasks were significantly higher (*p* < .001) when compared to the control, (Figures 4 and 5). In the suspended populations (Figure 5), there was a sustained increase in percentage of non‐viable amoebae throughout the rest of the experiment for both these treatments, in which all timepoints remained significantly different (*p* < .001) to the control. This trend is also seen for the non‐viable amoebae percentage in relation to the total population for MS‐222 and metomidate (Figure 4), with sustained, significantly higher percentages of non‐viable amoebae when compared to the control after day 8.

## 4 DISCUSSION

Parasitic diseases are a major bottleneck in salmonid aquaculture, with intense efforts to study the host–parasite interactions to find effective treatments. To obtain non‐lethal samples (sequential) from fish during an in vivo challenge, the use of anaesthetic is unavoidable (Zahl, Samuelsen, & Kiessling, 2012). Therefore, the first step taken towards establishing a non‐lethal sampling challenge model is to ascertain any inhibitory or assistive effect of the anaesthetic upon the chosen parasite. If any effect were to be present, the methodology would thus no longer be a true representation of the natural experimental parasite infection for each fish, detected immune responses, or pathology.

AQUI‐S^®^‐treated amoebae show rapid growth in total population in vitro from day 0 to 8 followed by a steady decrease in population as seen in the control (Figure 2). When comparing the suspended and attached population data (Figure 3), a relationship of AQUI‐S^®^ exposure and amoebae attachment is suggested. At day 12, mean numbers of attached amoebae in the controls drop to approximately one third of their day 8 mean numbers (from 296 ± 16 to 116 ± 14); concurrently, the control populations of amoebae in suspension rose by a similar amount (from 92 ± 16 to 254 ± 16). These data suggest that at day 12 there is a natural emigration of a substantial proportion of attached amoebae population into the seawater overlay. This movement was not observed in the AQUI‐S^®^ flasks, which showed significantly higher populations of attached amoebae at days 12 and 20, and sustained significantly lower amoebae populations in suspension from day 12 (Figure 3) although overall amoebae numbers (attached + suspended) remained similar between AQUI‐ S^®^ treatment and control. This infers that with repeated exposure to AQUI‐S^®^, an increased proportion of amoeba remain attached to their substrate; however, current AGD literature offers no hypothesis as to why this effect may be seen. During in vivo challenge experiments, an artificially elongated duration of attachment, during which parasitic amoeba could theoretically spend more time colonizing the gill substrate (Wiik‐Nielsen et al., 2016) than completing the natural emigration to the surrounding sea water, may lead to an increased level of disease progression and therefore an elevated immune response, which may not be comparable to the speed of disease progression found in the field. Nonetheless, with reported loss of virulence seen in cultured *N. perurans* possibly due to lack of attachment to gills (Bridle, Davenport, Crosbie, Polinski, & Nowak, 2015), increased attachment due to the use of isoeugenol‐based anaesthetics may help mitigate this problem, if similar attachment processes are involved.

In this study, the amoebae were classed as “non‐viable” primarily due to the lack of uptake of the Neutral Red vital stain (Repetto, Del Peso, & Zurita, 2008), but morphology was also taken into consideration. Amoebae in which no stain was seen all held the same spherical morphology (Figure 1), characteristic of in vitro cultures with a suboptimal subculturing schedule, suggesting this morphology is a response to overcrowding, lack of nutrients or environmental stressors (Lima, Taylor, & Cook, 2017; Wiik‐Nielsen et al., 2016). As the cultures in this study were washed regularly at 4‐day intervals, and percentage of non‐viable amoebae were higher in cultures with lower amoebae numbers (Figures 2 and 4), it is unlikely that the spherical morphology was caused by a build‐up of waste products from the amoebae themselves, or overcrowding, but more likely a response to anaesthetic exposure, or possibly limiting factors associated with the culture medium nutrients. The effect of the different anaesthetics, if any, on bacteria in the non‐xenic cultures, on which the amoebae may feed, was not recorded.

Recent work from Shijie et al. (2016) found that AQUI‐S^®^ at varying concentrations showed no significant effect on the viability or attachment capabilities of *N. perurans* 2 hr after single treatment. While working within the range of concentrations selected by Shijie et al. (2016) at 17 mg/L, this study was able to monitor viability of amoebae over a longer period (4 days after each treatment), allowing for a more comprehensive view of any possible impacts. With the exception of day 20, where there was a small but significant (*p* < .05) rise in the percentage of non‐viable amoebae in the total population compared to control (Figure 4), AQUI‐S^®^‐treated amoebae remained statistically similar to those counted in the control, with non‐viable amoebae remaining a small proportion (<10%) of the total amoebae population. This suggests that repeated exposure of AQUI‐S^®^ has no significant effect on the viability of *N. perurans*.

However, when comparing percentages of non‐viable amoebae as part of the suspended population, significant differences were found between the control and AQUI‐S^®^ populations at days 4, 20 and 28 (Figure 5), but as discussed above this reflects greater numbers of amoebae remaining attached in the AQUI‐S^®^ flasks, resulting in non‐viable amoebae forming a higher percentage of total suspended cells.

After a single exposure timepoint MS‐222, metomidate‐based anaesthetics seem to have a strong inhibitory effect upon both the growth of attached in vitro *N. perurans* cultures and a detrimental effect on viability of floating‐form amoeba after repeated exposure. As previously highlighted, the suspended amoebae in metomidate and MS‐222 flasks were in contact with their anaesthetics for substantially longer than required for in vivo anaesthetization. This increase in exposure time must be taken into consideration when evaluating the outcomes of the attached amoeba growth, as both populations are interdependent (Crosbie et al., 2012). However, it should also be considered that during an individually monitored challenge, fish are sampled with an in‐tank anaesthesia methodology (Collet et al., 2015), wherein the suspended amoeba will remain in contact with the anaesthetic while Stage 4 anaesthetized fish are netted out, processed and placed in a smaller recovery tank followed by the initial tank being drained with a flow‐through system. Any amoeba which remains in this tank after draining and refilling will have also been exposed to whichever anaesthetic was used for a longer duration that initially required for Stage 4 anaesthesia. Previous studies investigating adherence behaviour of *N. perurans* have shown high‐density colonization of aquarium surfaces, highlighting their potential as areas for attachment and replication (Rolin, Graham, McCarthy, Martin, & Matejusova, 2016) and may therefore act as an additional source of infection over time; however, the impact of amoebae shed from gills in reinfection and disease progression over the challenge, if any, is not known. It could be argued that this prolonged exposure of anaesthetics to the suspended amoeba population may even be more representative of the environmental conditions during non‐lethal sampling.

Metomidate is able to block the synthesis of plasma cortisol by inhibiting the mitochondrial cytochrome P_450_‐dependent enzymes required to catalyse the glucocorticoid (Small, 2003), an effect which has also been reported in fish treated with MS‐222 (Chevion, Stegeman, Peisach, & Blumberg, 1977; Fabacher, 1982). Akinrotimi, Gabriel, and Orokotan (2013) have shown that metomidate also has the dose‐dependent ability to impair the activities of plasma enzymes such as transaminases in the African sharptooth catfish, *Clarias gariepinus*, with the highest level of impairment seen at 12 mg/L. MS‐222 has been shown to inhibit the growth of Gram‐negative bacteria (Fedewa & Lindell, 2005); however, the concentrations (5,000–200 mg/L) used in this latter study were far higher than those used in vivo. Similar inhibitory effects on *N. perurans* p450 pathway and transaminases may have played a role in the suppression of population growth, attachment and viability of amoebae in the flasks treated with these anaesthetics (Figures 2, 3, 4 and 5). Such impacts may not be seen during in vivo challenges due to shorter exposure periods of fish to anaesthetics; therefore, the suitability of MS‐222 and metomidate as anaesthetics for non‐lethal sampling AGD challenges should be investigated further, with more efficient cleaning of the suspended population, utilizing faster spin times or filtering methods to obtain more appropriate exposure times.

In conclusion, this study illustrates the importance of selecting an appropriate anaesthetic when working with ectoparasites. Isoeugenol‐based, specifically AQUI‐S^®^, anaesthetics are suitable for both harvesting and repeated exposure in vivo and in vitro for work with the ectoparasite *N. perurans*.
